# Subjective Cognitive Concerns and Attitudes toward Genetic Testing Are Associated with Depressive Symptoms and Quality of Life after Genetic Testing for the Cerebral Cavernous Malformation Common Hispanic Mutation (CCM1)

**DOI:** 10.4236/jbbs.2020.102007

**Published:** 2020-02-25

**Authors:** Richard Campbell, Christine L. Petranovich, Savannah Cheek, Leslie Morrison, Blaine Hart

**Affiliations:** 1 Department of Psychiatry and Behavioral Sciences, University of New Mexico Health Sciences Center, Albuquerque, NM, USA; 2 Department of Rehabilitation Medicine, Children’s Hospital Colorado and the School of Medicine, University of Colorado Denver, Aurora, CO, USA; 3 University of New Mexico, Albuquerque, NM, USA; 4 Department of Neurology and Pediatrics, University of New Mexico Health Sciences Center, Albuquerque, NM, USA; 5 Department of Radiology, University of New Mexico Health Sciences Center, Albuquerque, NM, USA

**Keywords:** Quality of Life, Depression, Genetic Testing, Subjective Cognitive Concerns

## Abstract

**Purpose:**

This study aimed to characterize mood and quality of life and to examine the associations of these areas with subjective cognitive concerns and attitudes toward genetic testing for the Common Hispanic Mutation, a gene that has been associated with increased risk for CCM1.

**Method:**

Fifty-four adults with previous genetic testing for the Common Hispanic Mutation completed a mail survey that included assessments of the above identified areas.

**Results:**

Self-reported depressive symptoms and quality of life did not differ between those with positive and negative genetic test results. The negative group expressed a more favorable attitude toward genetic testing (*p* < 0.001). There was a trend toward more subjective cognitive concerns in the positive group (*p* = 0.06). Using generalized linear regression, more subjective cognitive concerns were associated with poorer quality of life and more depressive symptoms (*p* < 0.001). Poorer attitude toward genetic testing was also associated with poorer quality of life (*p* < 0.05).

**Conclusions:**

Subjective cognitive concerns and negative attitudes toward genetic testing may influence emotional well-being after genetic testing for the Common Hispanic Mutation. Additional research is needed that uses objective neuropsychological measures to understand the associations of subjective cognitive concerns, emotional well-being, and cognitive test performance in individuals with CCM1. There is also a need for research that focuses on protective factors and resiliency following genetic testing for CCM1 and the development of mental health interventions to preempt psychosocial difficulties.

## 1. Introduction

Cerebral cavernous malformation (CCM) is a rare neurological condition that is characterized by vascular malformations in the brain and spinal cord that can result in enlarged capillary channels, or caverns, and immature vessel walls. Broadly speaking, CCM is rare in the general population with estimates that range from 0.4% to 0.8% [1]. The familial variant, known as CCM1, is also rare, although more prevalent in parts of the western United States because of the Common Hispanic Mutation, an autosomal dominant mutation on chromosome 7q21-q22 [2]. Surgical resection is common and many patients with CCM have temporary or permanent neurological deficits, seizures, and chronic headaches [3]. In view of this risk for negative neurological sequalae, problems with cognition [4] and psychological functioning [5] may be expected, although existing CCM research has yet to verify this.

Given the genetic etiology and the potential for severe neurological problems, individuals who are known to be at-risk for CCM1 often undergo genetic testing. Existing research has not yet investigated the psychological implications of genetic screening in individuals who are at-risk for CCM1, although findings from other neurological medical conditions with a known or suspected genetic etiology may increase our understanding in this area. In an early study of Huntington’s disease, carriers, compared to non-carriers, reported decreased general psychological well-being in the initial 7 – 10 days and at 6 months after testing. However, this dissipated with time and carriers and non-carriers did not significantly differ 1 year after the testing [6]. A subsequent study replicated these findings and similarly found that while carriers and non-carriers differed in short-term psychological distress, it did not persist long-term [7]. Considering the APOE gene that is associated with risk for Alzheimer’s disease, positive genetic testing was associated with increased health behaviors [8]. In a separate study, participants reported that genetic testing was helpful for making decisions regarding their personal affairs [9].

The University of New Mexico Health Sciences offers CCM-specific clinical services and patient supports and is recognized as a Center for Excellence in CCM care by the Angioma Alliance. Patient-engagement and community-based participatory efforts suggested that patients with CCM1 and their families frequently described concerns related to negative attitudes about genetic testing, which resulted in the unwillingness of some at-risk relatives to have testing completed. Patients also frequently expressed concerns about their own mental health and cognitive functioning. Speaking to the latter, there is a growing appreciation of the associations of subjective cognitive complaints, risk for cognitive decline, and depressive symptoms. In a systematic review, Mendonca and colleagues [10] concluded that older adults with subjective cognitive complaints do not show significant cognitive decline, although subjective cognitive complaints were associated with a significantly high risk of developing dementia. In a separate study, Heser and colleagues [11] found that subjective cognitive complaints mediated the association between depressive symptoms and dementia, suggesting that depression and subjective cognitive complaints may be important when considering risk for cognitive decline in older individuals. However, to our knowledge, research in this area has focused on mild cognitive impairment and dementia and has not extended to individuals with other neurological conditions that confer risk for cognitive problems.

Taken together, patient-engagement and community-based participatory efforts at the University of New Mexico, a CCM Center of Excellence, have qualitatively indicated patient concerns about the unwillingness of some at-risk relatives to have genetic testing and their cognitive and mental health functioning. Existing research in patients with mild cognitive impairment and dementia suggests that subjective cognitive complaints and depressive symptoms may elevate risk for cognitive decline, although these areas have not yet to be examined in individuals with other neurological conditions. Prior research in other patient populations with a known or suspected genetic etiology has offered insights into the psychological implications of genetic testing, although similar research has not been completed in individuals with genetic testing for CCM1. To address these gaps in the literature, this study utilized a retrospective survey of individuals who had genetic testing for CCM1. The specific aims were to characterize mood and quality of life and to examine the associations of these areas with subjective cognitive concerns and attitudes toward genetic testing. It was hypothesized that a positive test result for the CCM1 mutation would be associated with more depressive symptoms and poorer quality of life. It was also expected that more self-reported cognitive concerns and poorer attitudes toward genetic testing would be related to more depressive symptoms and poorer quality of life.

## 2. Method

### 2.1. Recruitment

Procedures were approved by the institutional review board (IRB) at the institution that the study was completed. Data collection for this survey study was completed from October 2016-January 2018. Eligibility criteria included being 18 years of age or older, not being incarcerated at the time of participation, and English as the participant’s primary language. Potentially eligible participants were sent a recruitment letter by mail and then received a maximum of three follow-up phone calls within two weeks. If the individual agreed to participate, consent was obtained by phone and a survey packet was mailed to the home. An addressed and stamped envelope to the return the survey was provided. Estimated time for completion of the survey was one hour. Once the packet was returned, the participant received a $25 merchandise gift card for their time and effort. If the packet was not returned within two weeks, one follow-up reminder phone call was made.

### 2.2. Measures

As provided in Appendix 1, the investigators developed the Attitudes toward Genetic Testing questionnaire with a higher score reflecting a more favorable attitude. This questionnaire was largely based on community-based participatory activities with patients and their families; other questions were adapted from items on the Satisfaction with Decision Scale [12]. Responses were based on a Likert Scale and the total scores from these measures were used in the analyses that are described below.

Participants also completed the Centers for Epidemiological Studies—Depression Scale (CES-D) [13] as a measure of depressive symptoms. This measure was reported as a raw score with a score > 16 indicative of elevated depressive symptoms. Participants also completed the Quality of Life Inventory (QOLI) [14] as a standardized measure of overall well-being and life satisfaction. Scores from the QOLI were reported as T-scores (mean = 50, standard deviation = 10). From the National Institutes of Health Patient Reported Outcomes Measurement Information System (PROMIS), participants completed the Applied Cognition-General Concerns-Short Form 8A. This was reported as a raw score. All of these measures were selected as they have high levels of reliability and validity in assessing their respective constructs.

### 2.3. Statistical Approach

Mean group differences (positive versus negative) for the CES-D, QOLI, Attitudes toward Genetic Testing, and PROMIS Cognitive Concerns were reported. Generalized linear regression models were calculated with the CES-D and QOLI as separate dependent measures. Demographic control variables (gender and self-reported income), PROMIS Cognitive Concerns, and Attitudes toward Genetic Testing were used as independent variables. For each regression model, Variance Inflation Factor (VIF; *i.e*., ratio of the obtained model variance to that of a model with only one factor) and tolerance were calculated to assess for multicollinearity among main effects.

## 3. Results

### 3.1. Survey Response

Participants were randomly selected from a pool of 352 individuals with genetic testing for CCM1 as part of a previous study [15]. From that pool, recruitment was attempted for a total of 182 participants (112 positive and 70 negative). Questionnaires were received from 54 individuals, 37 of which had a positive and 17 had a negative test result. The 80% binomial confidence interval response rate was 25.5% - 34.1%. The most common barrier to recruitment was outdated contact information that resulted in an inability to make contact.

### 3.2. Sample Characteristics

Demographic and health-related information is reported in Table 1. In both groups, a large proportion of the participants were female, married, and had some college experience (e.g., some college, undergraduate, or graduate degree). The positive and negative groups did not differ in the time from genetic testing to survey completion, self-reported income, age, marital status, gender, community type, or highest level of education (*p* > 0.05). As would be expected, the positive group reported significantly higher annual CCM-related financial expenses and a larger proportion had a history of seizures, chronic headache, hemorrhage, and CCM-related neurosurgical intervention (*p* < 0.05).

### 3.3. Bivariate Correlations

The bivariate correlation between CES-D and QOLI was −0.67. It was −0.12 for PROMIS Cognitive Concerns and Attitudes toward Genetic Testing. The correlations of the predictors with the dependent measures ranged from −0.14 (CES-D and Attitudes toward Genetic Testing) to 0.71 (CES-D and PROMIS Cognitive Concerns).

### 3.4. Depressive Symptoms, Quality of Life, and Associated Factors

As reported in Table 1, the groups did not significantly differ in mean depressive symptoms (CES-D) or quality of life (QOLI). The negative group expressed more favorable attitudes toward genetic testing (*p* < 0.001). There was a trend toward more cognitive concerns (PROMIS Applied Cognitive Concerns) in the positive group, although this was above the threshold for statistical significance (*p* = 0.06).

Using generalized linear regression and considering the QOLI as the dependent variable, VIF and tolerance values were acceptable (*i.e*., tolerance > 0.1 and VIF ≤ 10) [16]. In this model, more subjective cognitive concerns (*p* < 0.001) and poorer attitudes toward genetic testing (*p* = 001) were associated with poorer quality of life, irrespective of test result (See Table 2).

Considering the CES-D as the dependent measure, VIF and tolerance values were again acceptable. For this model, lower self-reported income (*p* = 0.002) and more subjective cognitive concerns (*p* < 0.001) were associated with more depressive symptoms, again irrespective of test result (See Table 2).

## 4. Discussion

This study examined depressive symptoms and quality life in individuals with prior genetic testing for CCM1. Contrary to expectations, participants in both groups reported few problems in these areas. Minimal longer-term emotional distress is consistent with studies of those who had genetic testing for Huntington’s disease [6] [7] and the BRCA 1/2 mutations [17]. Prior studies of individuals who were at-risk for Huntington’s disease found that psychological distress after genetic testing dissipated with time [6] [7]. It is therefore notable that participants in this study completed the survey at an average of 4.8 years after genetic testing was completed. More problems may have been reported if the survey was completed closer to the time of genetic testing. As an additional consideration, many of the participants in this sample were connected to CCM-specific clinical programs, which may have protected against mood and quality of life issues.

Additional aims were to examine the associations of mood and quality of life with self-reported cognitive concerns and attitudes toward genetic testing. Consistent with expectations, more subjective cognitive concerns were associated with poorer quality of life and more depressive symptoms. In view of the existing literature that focused on cognitive decline in older individuals [11], the association of subjective cognitive concerns and depressive symptoms may pose elevated risk for cognitive decline in our sample. Concerns about cognitive deterioration and the potential outcomes of that, such as the possibility of decreased independence, work-related/occupational issues, and underlying disease progression, likely carry psychological burden and distress. In view of CCM-related neurological complications generally, close monitoring and support are warranted from a neuropsychological perspective. This may be especially true for patients with subjective cognitive concerns as this may further elevate their risk for objective cognitive difficulties and problems with mood and quality of life.

Our findings further indicated that a less favorable attitude toward genetic testing was associated with poorer quality of life. Considering this finding, it is unclear if a negative attitude was specific to genetic testing or CCM1 more broadly. That being said, it is interesting that quality of life was unrelated to a positive or negative test result. Potentially relevant, this study was comprised of individuals who were at-risk for CCM1. As such, many participants in both groups had relatives that experienced adverse medical events related to CCM1. Given this impact of CCM1 on the broader family system, the participants’ personal genetic test result may have little impact on overall perception of CCM1 and, in turn, quality of life.

Consistent with the broader literature, lower income was associated with more depressive symptoms, likely reflecting the negative impact of financial hardship on emotional wellbeing. However, this relationship may be more complex than income alone. Health economics research suggests that the association of income and depression decreases when other sociodemographic variables, such as employment status, are considered [18]. Socioeconomic factors may be particularly important for patients with chronic medical conditions, where medical expenses are often high and there may be work-related barriers, such as frequent medical appointments.

There are notable limitations of this study, including a small sample size and reliance on retrospective report after the genetic testing was completed. The response rate may be an additional limitation that impacts the generalizability of these findings to the larger population of individuals with CCM1 and rare neurological conditions more broadly. That being said, the response rate for this study was consistent with that of other healthcare-related mail surveys [19]. As an additional limitation, this sample may represent a specific group of individuals. As many of the participants shared a similar geographical location, there may be cultural influences on how emotional difficulties were viewed and reported. Given these limitations, the generalizability of these findings to the larger population of patients with sporadic/non-familial CCM and other neurological conditions with a genetic etiology is unknown. Specific to the findings with respect to subjective cognitive complaints, this study did not include objective, performance-based neuropsychological testing. It is therefore unknown if these self-reported cognitive complaints reflected brain-based deficits or only worries about cognitive functioning. As a final limitation, although information regarding CCM-related health status was reported, we did not collect information regarding overall current health status; this may be relevant when considering mood and quality of life.

Despite these limitations, this study has notable strengths. CCM is a rare condition and there is currently a dearth of research in this patient population. To our knowledge, this is the first study to examine emotional functioning in patients with CCM. Although this sample represented a select patient group, these findings nonetheless shed light on the emotional well-being and concerns of individuals who are at-risk for genetic conditions. These results also highlight multiple areas that may benefit from clinical attention, including that individuals who are at-risk for CCM1 may have subjective cognitive concerns and negative attitudes about genetic testing that influence their emotional well-being. From a services perspective, those considering genetic testing may benefit from psychosocial support. Individuals who have cognitive concerns and are at-risk for CCM1 may also benefit from a formal neuropsychological evaluation to better understand their current cognitive functioning and to assist with treatment planning. Finally, these results point to directions for future research, including a need for research that utilizes objective neuropsychological measures to understand the associations between subjective cognitive concerns, depressive symptoms, and cognitive functioning in individuals with CCM1. There is also a need for research that focuses on protective factors and resiliency following genetic testing for CCM1 and the development of mental health interventions to preempt psychosocial difficulties.

## Supplementary Material

Appendix 1

## Figures and Tables

**Table 1.** Sample characteristics and psychological data.

	Positive	Negative	*p*

N	37	17	--

Income	5% = less than $10,000 20% = $10 − 25,000 11% = 25 − 50,000 22% = 50 − 75,000 20% = 75 − 100,000 20% = more than $100,000	11% = less than $10,000 17% = 25 − 50,000 23% = 50 − 75,000 11% = 75 − 100,000 35% = more than $100,000	0.32
Age	50.81 (13.06)	48.35 (14.17)	0.54
Gender (% female)	78%	70%	0.53
Marital Status	78% = married 2% = divorced 14% = in relationship 5% = single	70% = married 5% = divorced 11% = in relationship 11% = single	0.81
Highest education	16% = high school or less 70% = college 10% = graduate 2% = not reported	17% = high school or less 70% = college 11% = graduate	0.32
Community type	24% = rural 37% = suburban 29% = urban 8% = not reported	35% = rural 35% = suburban 29% = urban	0.80
Time from genetic testing to survey	60.94 (21.08)	51.41 (24.20)	0.17
Annual CCM-related costs	$1767 (4522.87)	$9.37 (37.50)	0.04*
% with seizures	43%	11%	0.005*
% chronic headaches	59%	17%	0.004*
% history CCM-related neurosurgery	29%	0%	0.01*
% history of hemorrhage	54%	0%	0.003*
CES-D	13.91 (9.45)	12.05 (8.00)	0.45
QOLI	48.72 (11.75)	51.00 (9.82)	0.46
PROMIS Cognitive Concerns	21.51 (9.18)	17.31 (6.66)	0.06
Attitudes Toward Genetic Testing	45.47 (8.25)	54.88 (6.72)	<0.001**

*: Significant at 0.05 alpha level.

**: Significant at 0.001 alpha level.

**Table 2.** Generalized linear regression findings.

	QOLI		CES-D	
	*Unstd B*	*t* (*p*)	*Unstd B*	*t* (*p*)

Group	5.07	1.58 (0.12)	−3.69	−1.82 (0.07)
Income	1.08	1.24 (0.22)	−1.75	−3.30 (0.002)*
Gender	−4.13	−1.32 (0.19)	0.07	0.04 (0.96)
PROMIS Cognitive Concerns	−0.67	−4.16 (<0.001)**	0.61	6.12 (<0.001)**
Attitudes Toward Genetic Testing	0.42	2.46 (0.01)*	−0.17	−1.59 (0.11)

*: Significant at 0.05 alpha level.

**: Significant at 0.001 alpha level.
