# Supplementary material for: Subjective Cognitive Concerns and Attitudes toward Genetic Testing Are Associated with Depressive Symptoms and Quality of Life after Genetic Testing for the Cerebral Cavernous Malformation Common Hispanic Mutation (CCM1)
Source: J Behav Brain Sci. Author manuscript; Available in PMC 2020 May 28. (PMC7255433; doi:10.4236/jbbs.2020.102007)
Supplement: Appendix 1 [file NIHMS1578755-supplement-Appendix_1.doc]

Appendix 1. Attitudes about genetic screening questionnaire.

Please check the box that best describes your current opinions about genetic testing for the Common Hispanic Mutation.

|  | Strongly agree | Agree | Neither agree not disagree | Disagree | Strongly disagree |
| --- | --- | --- | --- | --- | --- |
| 1. My life is better because I had genetic testing. |  |  |  |  |  |
| 1. Overall, I am a happier person because I had genetic testing. |  |  |  |  |  |
| 1. My life is more stressful because I had genetic testing. |  |  |  |  |  |
| 1. My choice to have genetic testing has been stressful for my family members. |  |  |  |  |  |
| 1. Because I had genetic testing, my doctor better understands my healthcare needs. |  |  |  |  |  |
| 1. Overall, I am a healthier person because I had genetic testing. |  |  |  |  |  |
| 1. Since I was genetically tested, I feel hopeless because there is little that can be done about my health. |  |  |  |  |  |
| 1. I wish that I had not had the genetic testing. |  |  |  |  |  |
| 1. I feel angry because I had the genetic testing. |  |  |  |  |  |
| 1. Since I had genetic testing, I worry more about myself. |  |  |  |  |  |
| 1. Since I had genetic testing, I worry more about my family members, such as my children. |  |  |  |  |  |
| 1. The decision to have genetic testing was the best decision for me personally. |  |  |  |  |  |
| 1. My decision to have genetic testing is consistent with my personal values. |  |  |  |  |  |
